# WGCNA-based analysis of *MYL2* and its relationship with muscle fiber development during the embryonic stage in Inner Mongolia Albas White Cashmere Goats

**DOI:** 10.3389/fvets.2025.1658460

**Published:** 2025-08-26

**Authors:** Danni Wu, Xiaolong Xu, Dan Zhao, Qing Qin, Chongyan Zhang, Jiale Gao, Anru Xing, Qi Lv, Haijun Zhang, Zhihong Liu

**Affiliations:** ^1^Department of Animal Science, Inner Mongolia Agricultural University, Hohhot, China; ^2^Inner Mongolia Key Laboratory of Sheep & Goat Genetics, Breeding and Reproduction, Hohhot, China; ^3^Key Laboratory of Mutton Sheep & Goat Genetics and Breeding, Ministry of Agriculture and Rural Affairs, Hohhot, China; ^4^Erdos Agricultural and Animal Husbandry Science Research Institute, Ordos, China

**Keywords:** muscle fibers, WGCNA, Inner Mongolia Albas White Cashmere Goats, embryonic period, growth and development

## Abstract

The weight and quality of skeletal muscle are important indicators of meat product quality in livestock. The development of muscle fibers mainly occurs during the embryonic period, with the later stages of embryonic development being the critical time for muscle fiber formation. This stage determines postnatal growth rate, developmental traits in adulthood, and meat production performance. Therefore, studying the tissue structure and developmental patterns of muscle fibers during the embryonic period is crucial. This study utilized Weighted Gene Co-expression Network Analysis (WGCNA) to analyze the transcriptomes of goat skeletal muscle at 10 developmental stages during fetal development. A total of 10 co-expression modules were identified, with the blue module being selected for its relevance to embryonic development and muscle growth in the later stages of fetal development. Functional enrichment analysis revealed that this module is associated with mitochondrial processes (such as the tricarboxylic acid cycle and ATP synthesis) and is enriched in muscle-related pathways, including AMPK, Wnt, MAPK, and FoxO signaling pathways. The protein–protein interaction (PPI) network identified four key genes (*MYLPF*, *MYL2*, *PFKM*, and *TCAP*). The expression patterns of these four differentially expressed genes (*MYLPF*, *MYL2*, *PFKM*, and *TCAP*) identified in the network were validated through RT-qPCR, and the results showed a high consistency with the sequencing data. Immunohistochemical analysis confirmed that *MYL2* expression increased continuously, with the strongest positive staining observed in the cytoplasm during the later stages of muscle fiber development. These findings provide new insights into muscle fiber development and skeletal muscle growth in cashmere goats.

## 1 Introduction

Goats, as a major source of protein in developing countries, are estimated to number around 7.7 billion globally, with the majority concentrated in Asia and Africa (90%) (1). Goats are highly adaptable to extreme environments, making them particularly suitable for sustainable red meat production (2). Goat meat is rich in iron, potassium, and essential amino acids, making it a high-quality source of meat that meets the nutritional needs of humans (3). The Inner Mongolia Autonomous Region is a key area for cashmere goat farming, and the cashmere goat industry has become an important part of the local economy. Due to the high demand for cashmere in the market, the economic value of cashmere goats has long been focused on cashmere production, which has led to the underappreciation of their excellent meat production traits as a dual-purpose livestock species. In fact, meat production performance is a critical factor in determining the economic benefits of cashmere goat farming, and the growth and development of skeletal muscle play a vital role in this process (4).

The development of skeletal muscle can be divided into two distinct stages. The first stage of skeletal muscle development occurs during early embryogenesis and is marked by an increase in muscle cell number. The second stage takes place later in growth, involving muscle fiber hypertrophy and fiber type transformation (5), driving muscle development into the proliferative phase. Muscle fiber composition is a key determinant of meat quality. During fetal development, fiber type transformation occurs in two phases: initially, most primary myotubes express type I MHC, while secondary myotubes express neonatal MHC; later, type II MHCs (IIa, IIx, IIb) emerge (6). At birth, muscle is predominantly composed of oxidative fibers. As growth progresses, the proportion of oxidative fibers declines while glycolytic fibers increase (7), following the transition pattern I ↔ IIa ↔ IIx ↔ IIb (8). Zhou et al. (9) analyzed the longissimus dorsi muscle in newborn and 12-month-old Black Tibetan sheep and observed a shift from oxidative to glycolytic fibers over time, which also influenced overall growth and development. Therefore, investigating gene expression profiles related to muscle fiber transformation during the late stages of goat embryonic development can offer important insights for advancing the goat meat industry. Myosin light chain (MYL) is a multigene family that plays a crucial role in regulating skeletal muscle development and muscle fiber activity. Current research indicates that MYL family members regulate the growth and development of muscle cells in goat skeletal muscle. Studies have shown that *MYL2* is not expressed during *in vitro* differentiation of mouse myoblasts, but is strongly expressed in mature muscle tissues. Knockout of *MYL2* results in severe embryonic or primary muscle growth defects, indicating its essential role in muscle maturation and function (10). Predicted transcription factor binding sites (*MEF2*, *MyoD*, *MyoG*) in its promoter suggest *MYL2* plays a regulatory role in muscle cell proliferation and differentiation, highlighting its importance in myogenic lineage specification (11).

As research into skeletal muscle growth and development deepens, multiple signaling pathways have been identified as key regulators in livestock and poultry myogenesis, with Wnt, FoxO, AMPK, and MAPK pathways playing central roles. The Wnt pathway is crucial during both embryonic and postnatal muscle formation (12), and maternal nutrition can influence fetal mesenchymal stem cell differentiation via Wnt/*β*-catenin signaling, thereby affecting offspring muscle growth and meat quality (13). Studies have shown that Wnt11 regulates myofiber elongation in chicken embryos (14), Wnt5a promotes myogenic differentiation in cattle (15), and *β*-catenin enhances slow-twitch fiber expression in pigs, indicating fiber type-specific functions of Wnt signaling (16). FoxO1 regulates the coordinated differentiation of muscle and fat through the TGF-*β*/TGFBI pathway (17). AMPK, as a key metabolic regulator, influences fiber type conversion and mitochondrial function via mTOR and PGC1α signaling, contributing to slow-twitch fiber development in horses and mitigating muscle atrophy in mice (18, 19). The MAPK pathway, significantly upregulated in fetal Ujumqin sheep, may contribute to fat deposition and has been implicated in rapid development of myogenic cells during mid-to-late gestation in goats through Ras/Raf/Mek/Erk signaling (20, 21).

Weighted Gene Co-expression Network Analysis (WGCNA) is a transcriptomics-based approach that clusters genes with similar expression profiles into modules, correlates these modules with specific phenotypic traits, and facilitates the identification of key regulatory genes involved in complex biological processes (22). Although WGCNA has proven effective in various fields, its application to skeletal muscle development during goat embryogenesis remains underexplored. In this study, we employ WGCNA to systematically analyze the skeletal muscle transcriptomes of goats across ten distinct fetal developmental stages, aiming to uncover gene networks and regulatory modules associated with muscle fiber formation and differentiation. This approach enables the identification of core regulatory genes driving muscle fiber type transitions, thereby offering new insights into the molecular mechanisms underlying fetal muscle development. This research not only lays a theoretical foundation for a deeper understanding of the growth and development mechanisms of goat skeletal muscle but also provides strong support for molecular breeding as well as the development and utilization of genetic resources for muscle traits in specialized livestock species.

## 2 Materials and methods

### 2.1 Selection of experimental animals

Inner Mongolia Albas White Cashmere Goats from the Yiwei Cashmere Goat Breeding Farm in Ordos were utilized as experimental subjects in this experiment. All ewes used for sample collection were healthy, multiparous, and of similar age. Does were fed regularly and quantitatively according to their weights. The Offspring of these does (fetal goats specifically) were used as experimental animals. Biceps femoris muscle samples were collected every 10 days from gestational E45-E135, resulting in 10 sampling periods. Each period included three individuals per group for biological replicates. Collected samples were stored in 4% paraformaldehyde or liquid nitrogen for subsequent analysis.

### 2.2 Detection of paraffin sections and fast/slow-twitch fibers at different developmental stages

First, the samples were rinsed with running water to remove residual 4% paraformaldehyde. Paraffin blocks were then prepared through gradient alcohol dehydration, xylene transparency treatment, and liquid paraffin embedding. After slicing at a 5-μm thickness using a microtome, the sections were spread in a 45°C water bath and attached to slides. They were dried at 62°C and dewaxed with xylene. To reduce endogenous interference, the sections were sealed with a 3% H₂O₂/PBS solution (Servicebio) for 15 min at room temperature. This was followed by heat-induced antigen retrieval with citrate buffer (Servicebio) and blocking of nonspecific binding sites using 10% goat serum for 30 min. Next, the fast-twitch fibers antibody (1:400, Sigma M4276) and the slow-twitch fibers antibody (1:500, Sigma M8421) were diluted in 100-μL drops and incubated overnight in a wet box at 4°C. In the following day, an HRP-labeled secondary antibody was applied for reaction at room temperature for 1 h. After DAB chromogenic staining (Servicebio), the nuclei were counterstained with hematoxylin for 30 s. Then, the sections were dehydrated through gradient alcohols and sealed with neutral gum. Section images were captured using a Zeiss MRC5 inverted fluorescence microscope equipped with the ZEN 3.1 Image Acquisition System. For each sample, two to three sections were randomly selected, and three non - overlapping fields were chosen from each section. The counts of fast and slow - twitch fibers were quantitatively analyzed via ImageJ software. Data are presented as the mean ± standard deviation; one-way ANOVA was performed using SPSS version 22, where *p < 0.05* indicated significance.

### 2.3 RNA extraction, cDNA library construction, and RNA sequencing

Transcriptome sequencing was conducted on fetal biceps femoris samples from Inner Mongolia Albas White Cashmere Goats (45–125 days, *n* = 3/group at 10-day intervals). Total RNA was extracted using Trizol reagent, and its purity was assessed by Nanodrop 2000 (A260/A280 values between 1.8 and 2.1; A260/A230 values > 2.0). RNA integrity was verified using the ChemiDoc XRS Gel Imaging System (Bio-Rad, USA), ensuring a clear display of 18 s/28 s rRNA bands with an RIN ≥ 7.0. After enriching mRNA from quality-compliant samples, a cDNA library was constructed with the TruSeq RNA Library Preparation Kit (Illumina). Paired-end 150 bp sequencing was performed on the Illumina HiSeq 2000 (Thermo Fisher Scientific, USA) platform to obtain at least 6 Gb of data per sample. FastQC software was used to evaluate the sequencing data quality; Trimmomatic software was used to remove low-quality sequences; HISAT2 software was used to align reads to the goat reference genome; and DESeq2 software was used to analyze gene expression differences.

### 2.4 WGCNA analysis of phenotype-related modules

WGCNA is a method for identifying gene modules significantly associated with specific phenotypes. In this study, we utilized the WGCNA R package (version 3.2.2) to analyze the relationship between co-expressed gene clusters and skeletal muscle fiber development phenotypes. The analysis used the Topological Overlap Matrix (TOM) to quantify gene associations (23, 24). Gene modules were categorized based on the sparsity of inter-gene connections, and the TOM matrix was transformed into a dissimilarity matrix to create a hierarchical clustering tree. The required gene modules were generated using a dynamic hybrid cutting algorithm, with the minimum merging distance set at 0.25 (25, 26). In the module detection process, each setting must have at least 30 genes. Different colors were used to identify various gene types in the resulting multiple gene modules. Pearson correlation analysis was then conducted to assess the correlation strength between the eigenvectors of each module and the experimental groups (with *p* values indicating significance) to identify modules significantly related to biceps femoris development. Finally, a heat map was used to compare gene expression trends across different groups, and a specific heat map for the core gene expression patterns corresponding to the target module was created to illustrate their expression levels.

### 2.5 Functional enrichment analysis of gene modules associated with muscle fiber growth and development

In the module related to muscle fiber development, genes with *p < 0.05* and |log₂FC| > 1 were identified as differentially expressed genes (DEGs). GO annotations^1^ and KEGG pathway enrichment analysis^2^ were performed using the Lianchuan Biological OmicStudio cloud platform to explore the functions of these genes through cluster analysis. The GO functional enrichment analysis included cell component (CC), molecular function (MF), and biological process (BP). Bar and bubble charts were used to provide an intuitive identification of biological processes linked to key genes in target modules, along with associated metabolic and signal transduction pathways. This visual analysis enhances our understanding of the potential roles of these DEGs in muscle fiber development.

### 2.6 Screening of key genes and construction of a PPI network

The top 50 module hub genes were queried in STRING (27)^3^ with medium confidence (0.4) for PPI predictions. Cytoscape (28) (v3.10.1) was used to visualize the preliminary networks, and node importance analysis was conducted via the CytoNCA plugin. Key genes were prioritized based on connectivity strength and expression level, and then re-analyzed in STRING (confidence 0.4) for validation. The final PPI networks were reconstructed in Cytoscape to illustrate the direct regulatory relationships among core genes.

### 2.7 Use of RT-qPCR to verify the expression level of target genes

**Figure 1:**
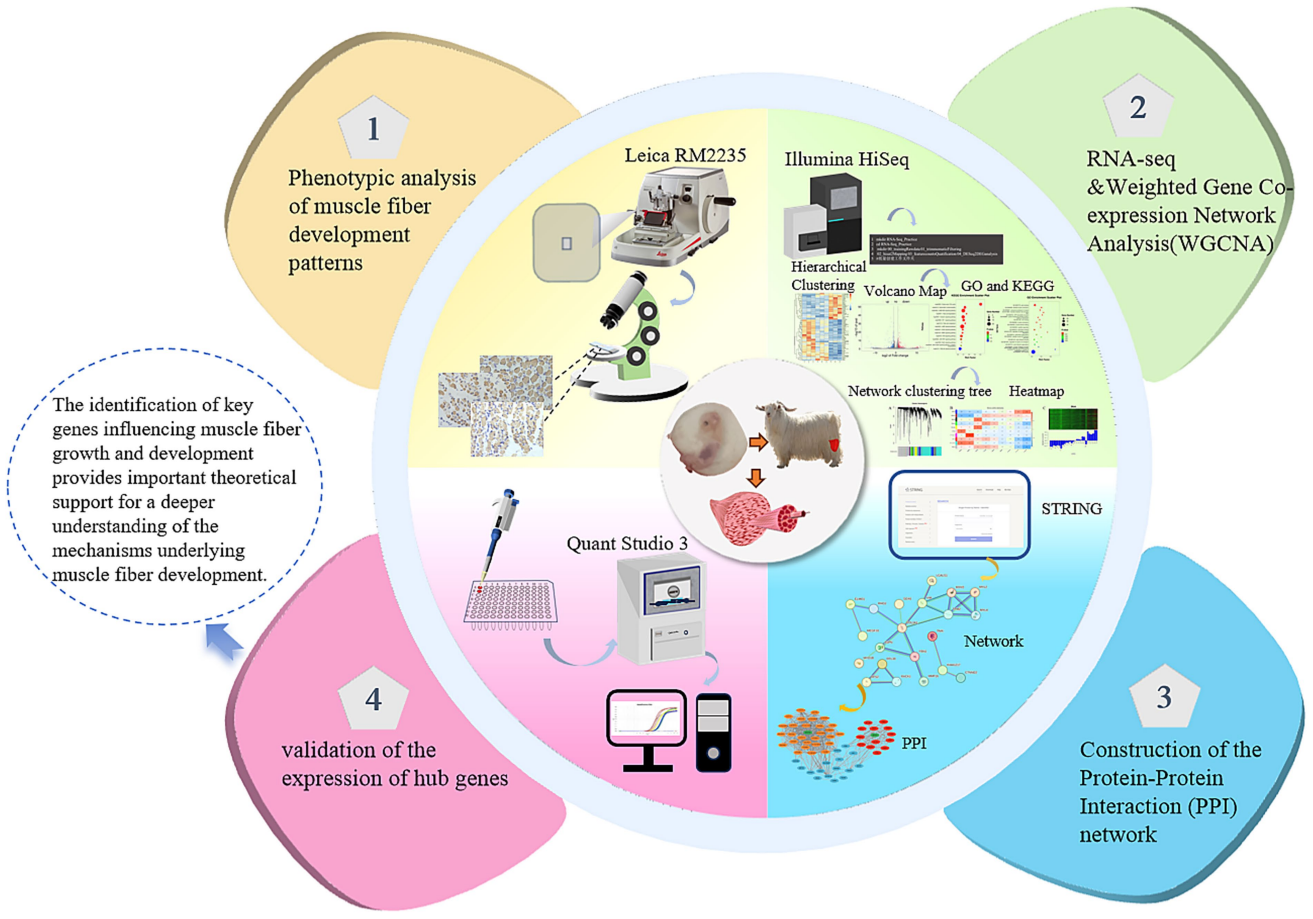
Topological four-leaf clover diagrams of different chapters.

To verify the repeatability and accuracy of muscle samples at different growth stages, qPCR was performed on 4 key genes. RNA samples meeting quality criteria (RIN ≥ 7.3, A₂₆₀/A₂₈₀ > 1.8, A₂₆₀/A₂₃₀ > 2.0) were reverse-transcribed to cDNA using a Takara kit. Primer 5.0 was used to design specific primers for the target genes and the internal reference gene *GAPDH*, yielding 4 primer pairs (Table 1). The qPCR used a 10-μL reaction volume: 5 μL of 2X TB Green Premix Ex Taq II (TIi RNaseH Plus), 0.2 μL Rox mixture, 0.8 μL forward/reverse primers (10 μM), 1 μL cDNA template, and 3 μL ddH₂O. Three biological replicates were set up before amplification (95°C pre-denaturation for 10 min → 40-cycle amplification → melt-curve analysis). The relative expression levels were calculated using the 2^-ΔΔ^Ct formula (29, 30), normalizing both target gene and *GAPDH* expression. GraphPad Prism 7 was used to compare qPCR and RNA-sequencing results and generate visual charts (see Figure 1).

**Table 1:** Primer sequences.

Primer name	Login number	Primer sequence (5′ → 3′)	Annealing temperature (°C)	Length (bp)
GAPDH	XM_005680968.3	F:CGGCACAGTCAAGGCAGAGAAC	61.45	22
		R:CACGTACTCAGCACCAGCATCAC		
MYLPF	NM_001285754.1	F:GGCGGAAGCTCCAATGTCTT	58.3	82
		R:CCATGGCTGCAAAAGTGTCC		
MYL2	XM_005691447.3	F:GAGCAGATGTTCGCCGCCTTC	64.21	21
		R:TTTCTTCTCCGTGGGTGATGATGTG		
PFKM	XM_018047861.1	F:GACACTGCCCTCAACACCATCTG	60.82	23
		R:ATGAGCCGCCCATCGTTTCAATG		
TCAP	NM_001285752.1	F:CCAGCTGTCGGAGGAGAACT	59.6	20
		R:TGTGGACAGCGTCAGATCCT		

### 2.8 Expression of *MYL2* in different developmental stages of skeletal muscle

Five-micrometer skeletal muscle paraffin sections were dewaxed in xylene and blocked with 3% H₂O₂/PBS (Servicebio). Antigen retrieval was done using citric acid buffer (Servicebio), and then the sections were sealed with 10% sheep serum. The sections were incubated overnight at 4°C with the primary antibody and then for 1 h at room temperature with the HRP-labeled secondary antibody. DAB (Servicebio) staining turned positive areas brown, followed by 30-s hematoxylin counterstaining. After gradient ethanol dehydration and xylene clearing, the sections were coverslipped with neutral resin. Three sections per sample were imaged using a Zeiss MRC5 microscope (10 × objective, ZEN 3.1 Image system). Three non-overlapping fields per section were analyzed using ImageJ. One-way ANOVA was performed using SPSS 22.0 with a significance threshold of *p < 0.05*, and the data were expressed as “mean ± standard deviation.”

## 3 Results

### 3.1 Development pattern of biceps femoris in Inner Mongolia Albas White Cashmere Goat fetus

**Figure 2:**
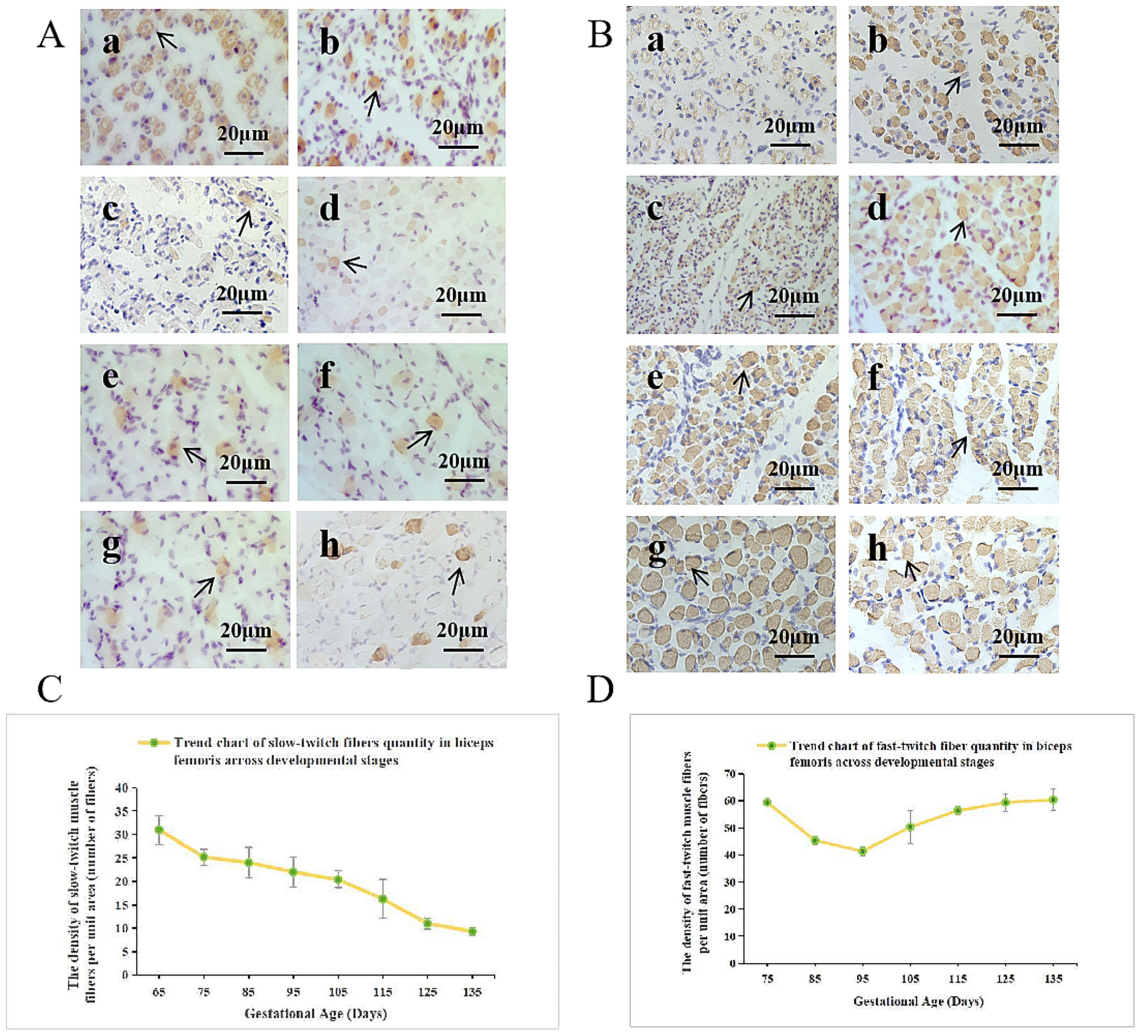
Developmental pattern of the biceps femoris in Inner Mongolia Albas White Cashmere Goat fetuses. **(A)** Slow-twitch fibers and **(B)** fast-twitch fibers were analyzed at embryonic stages E65 to E135 using immunohistochemistry (IHC, 40×). Images a–h correspond to E65, E75, E85, E95, E105, E115, E125, and E135, respectively. **(C)**Trend chart of slow-twitch fiber quantity in the biceps femoris across developmental stages. The x-axis represents the duration of pregnancy in days, while the y-axis indicates the density of slow-twitch muscle fibers per unit area. **(D)** Trend chart of fast-twitch fiber quantity in the biceps femoris across developmental stages. The x-axis represents the duration of pregnancy in days, while the y-axis indicates the density of fast-twitch fibers per unit area.

Based on the previous research results of the research group on embryonic immunohistochemistry, they identified the types of biceps femoris fibers in Inner Mongolia Albas White Cashmere Goat fetuses at various stages (31). Slow-twitch fiber staining (Figure 2A) revealed that at gestational day 65 (E65), muscle fibers showed a typical primary hollow structure with 100% slow-twitch fiber composition. From E65 to E135, slow-twitch fiber density progressively decreased. Fast-twitch fiber staining (Figure 2B) showed a decline from E75 to E95, followed by an increase from E95 to E135. Quantitative analysis (Figures 2C,D) has demonstrated that slow-twitch fiber content per unit area peaked at E65, then declined linearly until E135 and that Fast-twitch fiber density decreased from E75-E95, then increased to reach maximum at E135. These findings indicate a critical transition in muscle fiber composition during late embryonic development. The biceps femoris initially dominated by slow-twitch (type I) fibers, gradually shifting to fast-twitch (type II) fibers, coinciding with enhanced biomechanical demands for hindlimb movement.

Studies have demonstrated (32) that fast-twitch fibers exhibited higher myosin ATPase activity and enhanced glycolytic metabolism, which are closely linked to motor function adaptation. Therefore, systematic analysis of key candidate genes and their molecular mechanisms regulating muscle fiber type conversion during late embryonic development in Inner Mongolia Albas White Cashmere Goats is scientifically valuable for understanding skeletal muscle development.

### 3.2 Transcriptome data quality control

**Figure 3:**
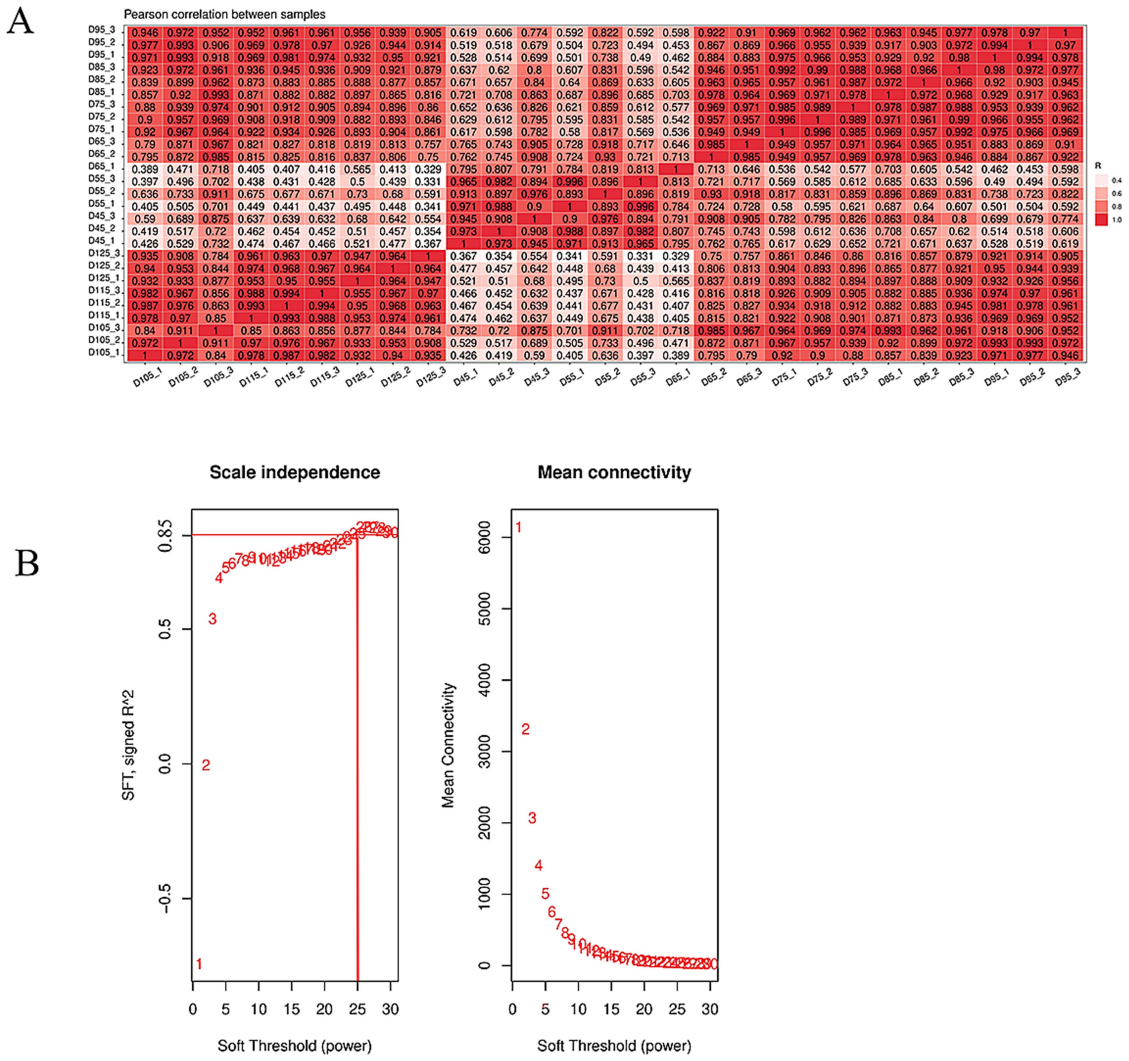
Quality control of transcriptome data. **(A)** The correlation analysis diagram of the transcriptome data further confirmed that the Pearson correlation coefficients among the samples were close to 1, reinforcing the conclusion that the sequencing results were reliable and ready for further examination. **(B)** WGCNA soft threshold selection used scale-free topological fitting (*R*^2^ = 0.85), determining *β* = 25 as the optimal soft threshold. The abscissa represents soft threshold values, while the ordinate depicts mean gene connectivity (adjacency function) at each *β*.

In this study, we sequenced 27 samples on the Illumina HiSeq 2000 platform, obtaining an average of 7.5 G of data per sample, with read counts ranging from 50,961,684 to 53,891,466. Reads with over 2 N bases were excluded. We removed low-quality ends and splice sequences, keeping only complete segments of ≥16 nt. We aligned the clean read segments to the goat reference genome using HISAT2, with an average alignment ratio of 95.13% (Table 2). A heat map of inter-sample correlation coefficients showed strong consistency among biological replicates (Figure 3A), validating the data for further analysis. Before WGCNA analysis, we filtered out genes with FPKM< 1 and those undetected in over 80% of samples, leaving 431,406 genes. We set a soft threshold of 25 (*R*^2^ = 0.85) through scale-free topological network fitting to construct a co-expression network for subsequent analysis (Figure 3B).

**Table 2:** The extracted effective RNA-seq sequences showed Pearson correlation coefficients close to 1 among samples, indicating high-quality sequencing data suitable for subsequent analyses.

Sample	Raw data	Clean reads	%	Q30%	GC content%
D45_1	50,917,640	49,119,652	96.47	97.77	48.50
D45_2	52,429,922	50,613,328	96.54	97.68	48.50
D45_3	51,801,624	49,949,658	96.42	97.77	48.50
D55_1	50,491,464	48,030,324	95.13	97.78	48.50
D55_2	52,170,316	50,030,278	95.90	97.71	49.00
D55_3	52,495,036	50,342,346	95.90	97.75	48.50
D65_1	51,217,364	49,356,978	96.37	97.68	49.50
D65_2	51,135,754	49,264,642	96.34	97.68	49.00
D65_3	54,571,120	52,548,474	96.29	97.72	48.50
D75_1	50,961,684	48,948,256	96.05	97.88	49.50
D75_2	51,289,190	49,203,572	95.93	97.92	49.50
D75_3	51,180,382	49,383,540	96.49	97.92	49.00
D85_1	51,992,816	49,479,936	95.17	97.90	49.50
D85_2	51,273,162	49,231,204	96.02	97.91	49.00
D85_3	53,891,466	51,730,668	95.99	97.92	49.00
D95_1	52,019,510	49,928,032	95.98	97.88	49.50
D95_2	51,112,304	49,089,104	96.04	97.93	49.50
D95_3	51,203,578	49,588,016	96.84	97.63	49.50
D105_1	51,001,988	49,414,682	96.89	97.77	49.50
D105_2	52,551,218	50,874,096	96.81	97.64	49.50
D105_3	53,225,506	51,560,860	96.87	97.65	49.00
D115_1	53,872,854	52,269,372	97.02	97.76	49.00
D115_2	51,095,816	49,488,306	96.85	97.67	49.50
D115_3	50,822,544	49,228,430	96.86	97.64	49.50
D125_1	50,309,986	48,531,076	96.46	97.67	49.50
D125_2	52,330,512	50,717,450	96.92	97.84	49.50
D125_3	50,467,724	48,751,146	96.60	97.93	49.50

### 3.3 WGCNA-screened modules related to skeletal muscle development

**Figure 4:**
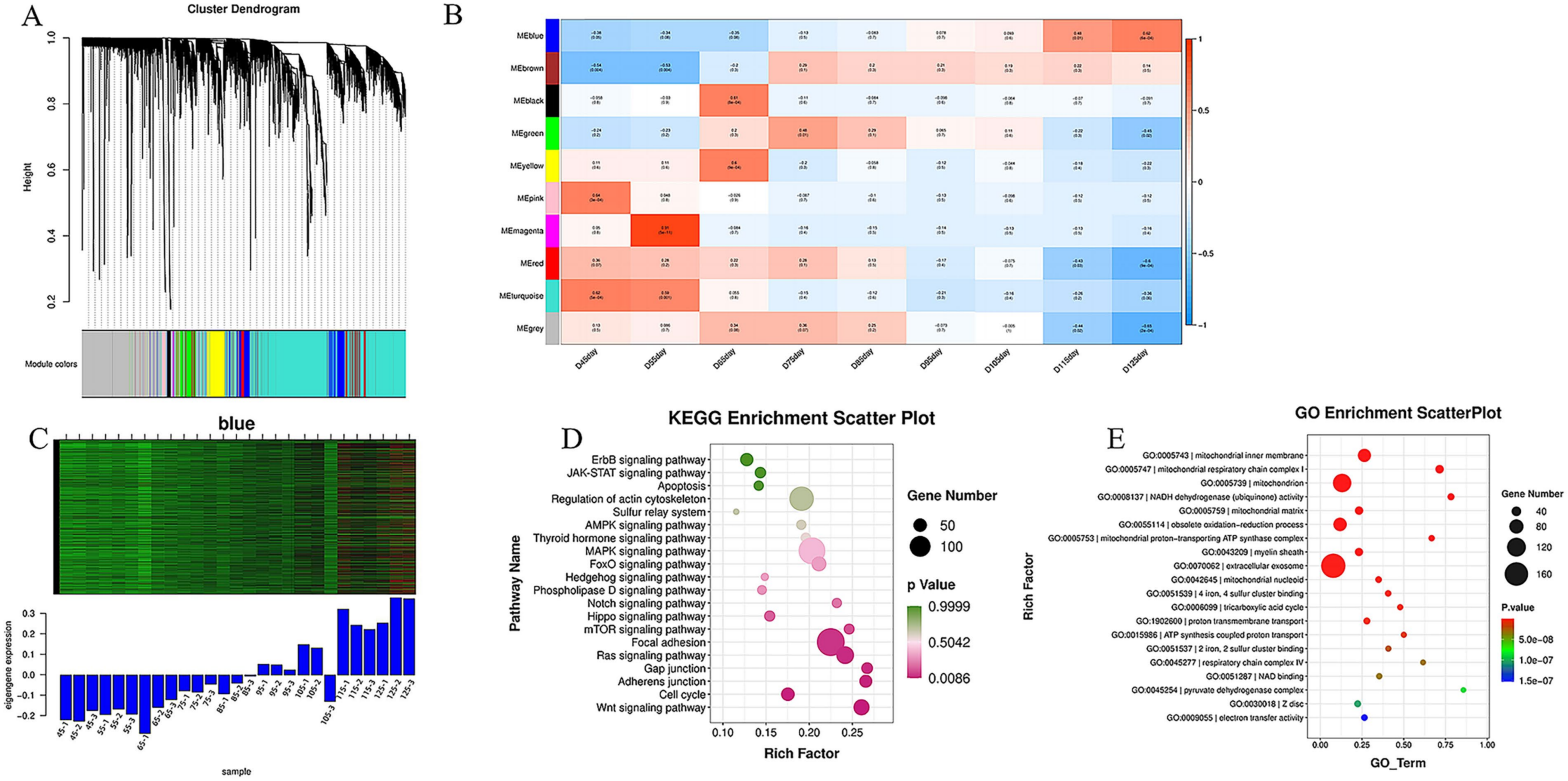
WGCNA screening and functional enrichment analysis of modules related to skeletal muscle development. **(A)** Gene co-expression network clustering dendrogram: colors represent distinct modules, resulting in 10 merged co-expression modules; **(B)** A correlation heatmap shows the relationship between each module and sample grouping. Values approaching 1 indicate a strong positive correlation (red), while values near −1 indicate a strong negative correlation (blue). The significance *p*-value is shown in parentheses; smaller values denote greater statistical significance. **(C)** Gene expression heat map for the blue module: the upper section displays gene expression levels, with red indicating higher expression and green lower expression across groups. The lower section presents a histogram of these gene expressions across various groups. **(D)** We identified genes linked to muscle fiber development within the blue module, generating a KEGG pathway enrichment dot plot revealing 26 enriched pathways. **(E)** GO functional enrichment analysis was performed on 1,204 genes from the blue module.

Using hierarchical cluster analysis of scale-free topological networks (scale-free *R*^2^ = 0.85), we classified 431,406 genes into 10 co-expression modules (Figure 4A). Temporal correlation analysis showed a significant dynamic relationship between the blue module and embryonic development: it had the strongest negative correlation at E45 and the strongest positive correlation at E125, reflecting the developmental progression from E45 to E125 (4) (Figure 4B). As the gene expression trend of this module matched the muscle fiber type transformation, we identified it as the core module for further analysis. We evaluated the gene expression levels in this module and created heat maps (Figure 4C). The results revealed that blue module genes were expressed at low levels during early embryonic stages (E45-E75) but were significantly up-regulated during later muscle fiber development (E95-E135), further confirming its association with muscle fiber development.

### 3.4 Results of GO and KEGG enrichment analysis of blue module genes

Functional enrichment analysis of the 1,204 genes in the blue module showed significant enrichment in the mitochondrial intima, respiratory chain complex I, tricarboxylic acid cycle, and ATP energy metabolism (Figure 4D). KEGG pathway analysis identified 26 pathways related to muscle fiber development, such as the AMPK, Wnt, MAPK, and FoxO signaling pathways. These findings highlight the crucial roles and regulatory mechanisms of the blue module in muscle fiber development (Figure 4E).

### 3.5 Use of PPI and co-expression networks to identify key genes related to muscle fiber growth and development

**Figure 5:**
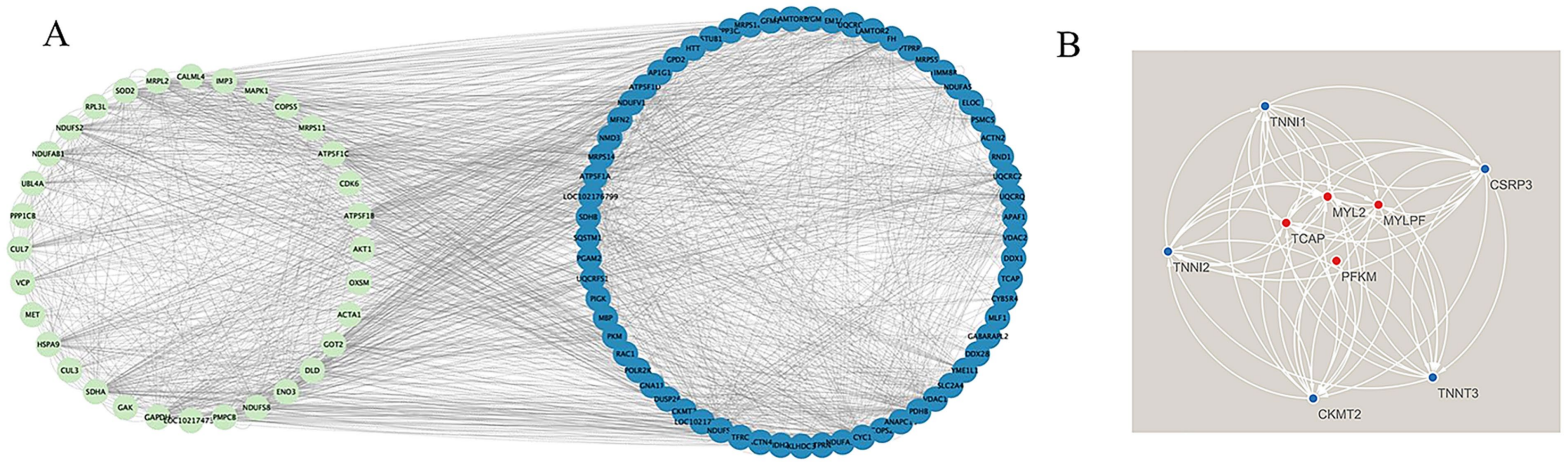
Construction of the PPI network and identification of hub genes. **(A)** Relationship map of the top 50 genes within the blue module; **(B)** Protein–protein interaction network illustrating four key genes in the blue module.

We constructed a protein–protein interaction (PPI) network for muscle fiber development in the blue module using the STRING database. The results showed that genes in this network mainly clustered into two major gene sets, including a myosin light chain (MYL) family related to muscle fiber development. We selected the top 50 genes based on connectivity within the module and created a gene network diagram (Figure 5A) with Cytoscape software to identify core genes. Four target genes (*MYLPF*, *MYL2*, *TCAP*, and *PFKM*) were identified as key for late-stage muscle fiber growth and development (Figure 5B; Table 3). These findings offer valuable insights into the molecular mechanisms of muscle fiber development.

**Table 3:** Criteria for key gene screening.

Gene	Enrichment of KEGG pathway	GO function	Expression ranking	Connectivity
MYLPF	4	4	2	407.38
MYL2	8	12	9,729	160.87
TCAP	0	21	31	417.14
PFKM	9	21	47	358.31

### 3.6 RT-qPCR verification of key gene expression content

**Figure 6:**
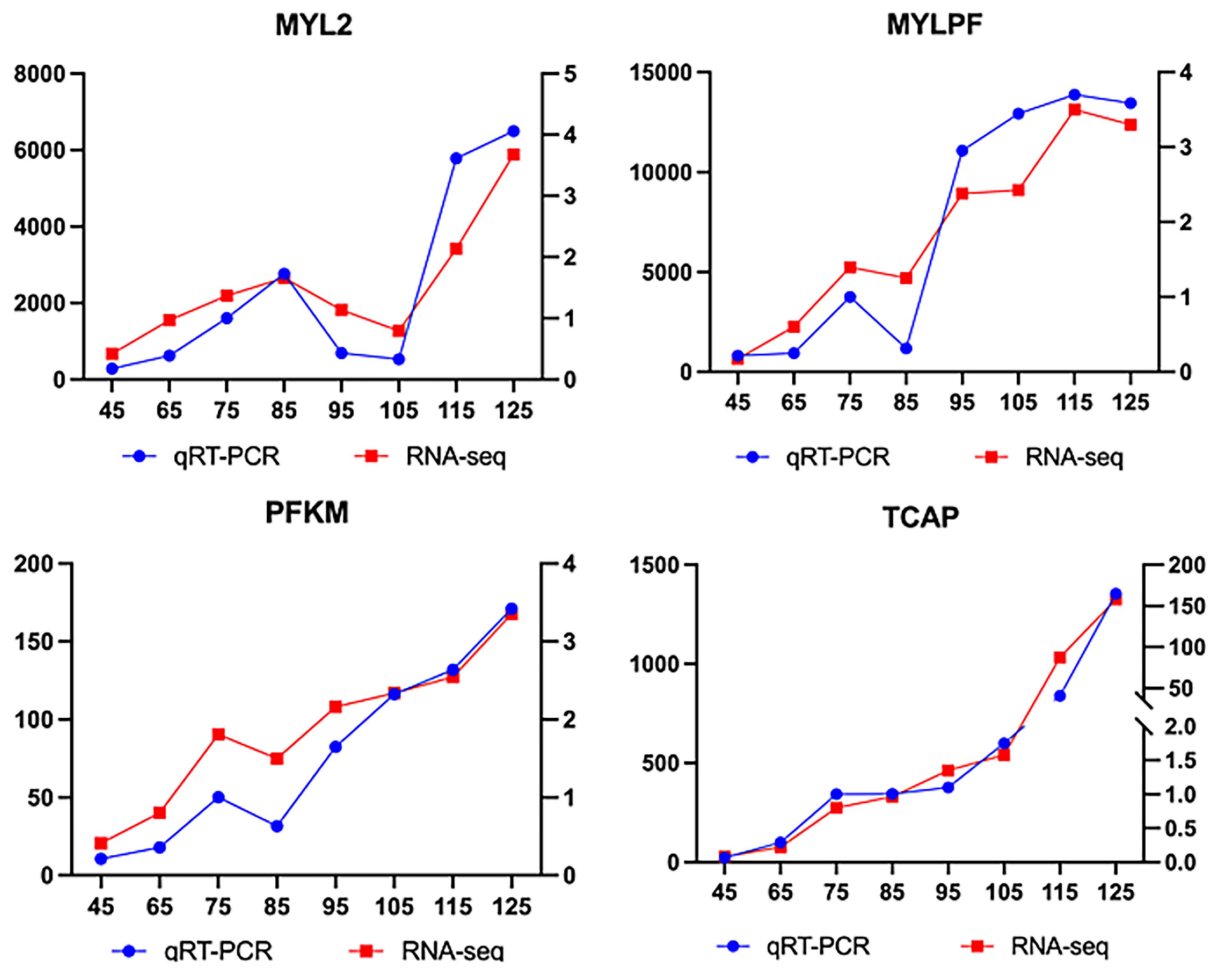
Comparative analysis of expression trends for key genes between RT-qPCR and RNA-Seq: results from the previous study 25.

In this study, four key genes (*MYLPF*, *MYL2*, *TCAP*, and *PFKM*) involved in muscle fiber development were validated using RT-qPCR. Time-series expression analysis revealed stage-specific regulation of these genes during biceps femoris development in Inner Mongolia Albas White Cashmere Goats (Figure 6). Early stage (E45-E75): All genes were significantly upregulated, with *MYLPF* (a fast-twitch fiber marker) showing the highest expression increase, followed by *PFKM* (a key glycolytic enzyme). Middle stage (E75-E95): *MYLPF* and the anchor protein *TCAP* maintained stable expression, while *MYLPF* and *PFKM* transiently downregulated at E85, indicating potential metabolic transitions. Late stage (E95-E125): *MYLPF* and *PFKM* remained upregulated, whereas *MYL2* displayed a “V-shaped” biphasic pattern: downregulation from E85 to E105, followed by upregulation from E105 to E125. These dynamic changes corresponded with muscle fiber maturation processes. Therefore, further investigation into *MYL2* gene expression across different periods was conducted.

### 3.7 Expression of *MYL2* in different developmental stages of skeletal muscle

**Figure 7:**
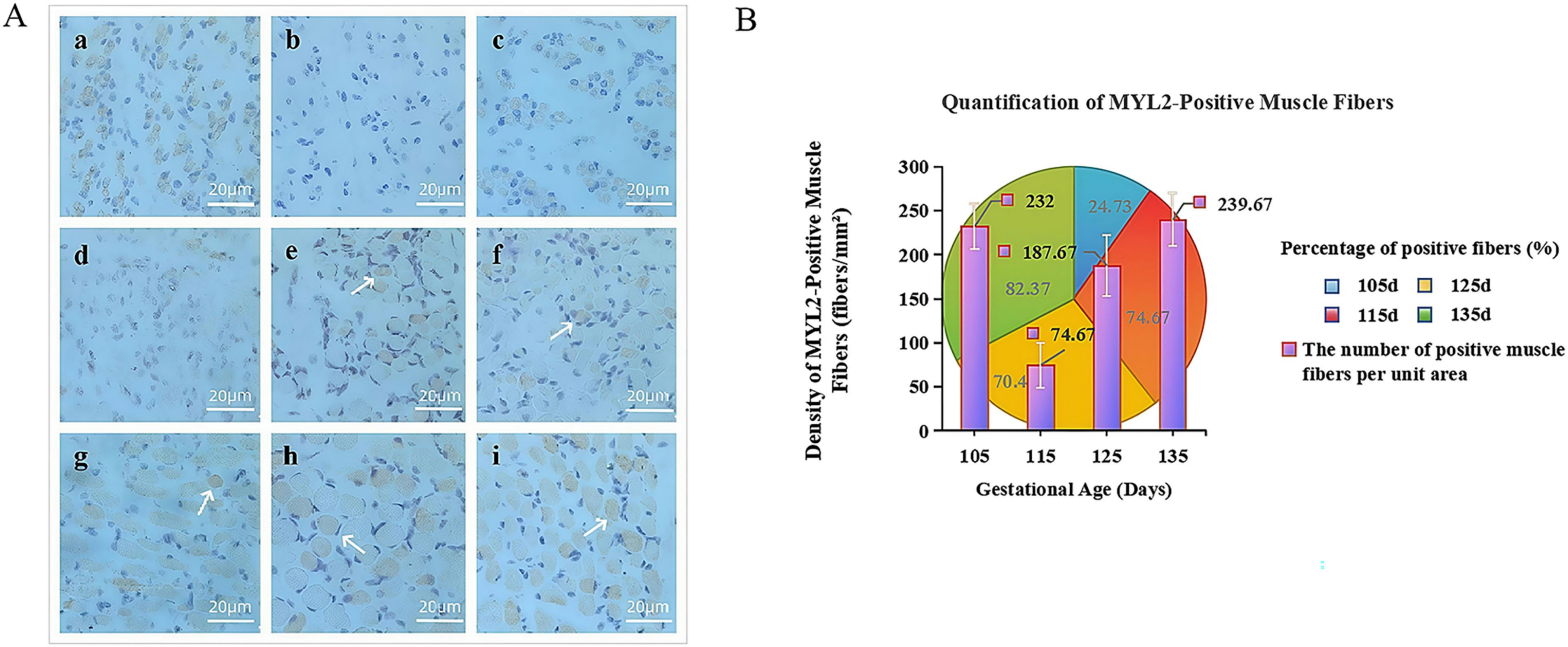
**(A)** Immunohistochemical comparison map of the *MYL2* gene in Inner Mongolia Albas White Cashmere Goats at 45 d, 65 d ~ 135 d. Groups a-i represent the biceps femoris during embryonic development (*MYL2*, 40×): a (E45), b (E65), c (E75), d (E85), e (E95), f (E105), g (E115), h (E125), and i (E135). **(B)** Trend chart of positive results for the *MYL2* gene. The x-axis shows gestation duration in days, while the y-axis indicates the number of positive muscle fibers per unit area. The pie chart displays the proportion of *MYL2* positivity across different periods, with colors representing positive rates on specific days. The histogram illustrates density of positive muscle fibers per unit area.

Based on our previous immunohistochemical study of *MYL2* gene expression during the development of the fetal biceps femoris in Inner Mongolia Albas White Cashmere Goats (23) (Figure 7A), significant *MYL2*-positive signals (DAB-stained yellow-brown) were detected in fetal samples at E45 and E75-E135. However, no specific expression was observed at E65. This absence might be because E65 is a critical transition period for muscle fiber type conversion, during which slow-twitch fibers gradually differentiate into mature fast-twitch fibers. As a fast-twitch fiber-specific gene, *MYL2* expression could be temporarily inhibited by upstream regulatory factors at this stage.

Based on the statistics of *MYL2*-positive muscle fibers in the late-stage development (Figure 7B), *MYL2* is primarily expressed in the cytoplasm. Muscle growth accelerated during this period. The number of positive muscle fibers was lowest at E115 and highest at E135, with the strongest signal at E135. This dynamic pattern of first decreasing and then increasing suggests that *MYL2* may regulate muscle fiber growth and development during the terminal differentiation stage of muscle fibers, influencing their growth processes.

## 4 Discussion

In recent years, WGCNA has emerged as a crucial bioinformatics tool in studying cancer pathogenesis and the regulation of plant and animal development. WGCNA clusters genes with similar expression patterns into functional modules, thereby revealing the cooperative regulatory relationships among gene groups within specific biological processes or signaling pathways (33). In this study, we applied WGCNA to a transcriptome dataset of 27 samples across various developmental time points. We analyzed the expression levels and trends of 15,978 effective genes and constructed 10 co-expression modules. Notably, genes in the blue module showed a transition from negative to positive co-expression correlation intensity, mirroring the biological process of skeletal muscle fiber differentiation and maturation. Thus, we chose the blue module for in-depth analysis.

Pathway enrichment analysis of the blue module identified 1,204 differentially expressed genes associated with 26 signaling pathways relevant to muscle fiber development. Notably, AMPK, Wnt, MAPK, and FoxO pathways exhibited significant enrichment. Additionally, GO functional analysis revealed that these genes mainly participate in mitochondrial respiratory chain-related biological processes. Mitochondria are essential for oxidative phosphorylation; they transfer hydrogen and electrons to the ATPase complex via inner membrane enzymes, consuming oxygen and generating energy (34). ATP is vital for various bodily functions and serves as the direct energy source during muscle contraction; thus, its synthesis efficiency directly affects skeletal muscle metabolism (35, 36) The blue module was highly enriched with genes involved in mitochondrial ATP synthesis, indicating its essential role in muscle fiber development. Notably, the biceps femoris, characterized by high meat production potential, showed the most significant enrichment of oxidative phosphorylation-related genes, consistent with its elevated energy requirements for muscular function. While mitochondria play a crucial role in ATP synthesis and influence skeletal muscle metabolism, several factors regulate this process. Besides key glycolytic and aerobic enzymes, maintaining mitochondrial structural integrity is essential for efficient ATP production (37). The Wnt signaling pathway regulates embryonic muscle development by promoting myoblast differentiation and fusion through mechanisms guiding stem cell differentiation and multinuclear muscle tube formation (38).

**Figure 8:**
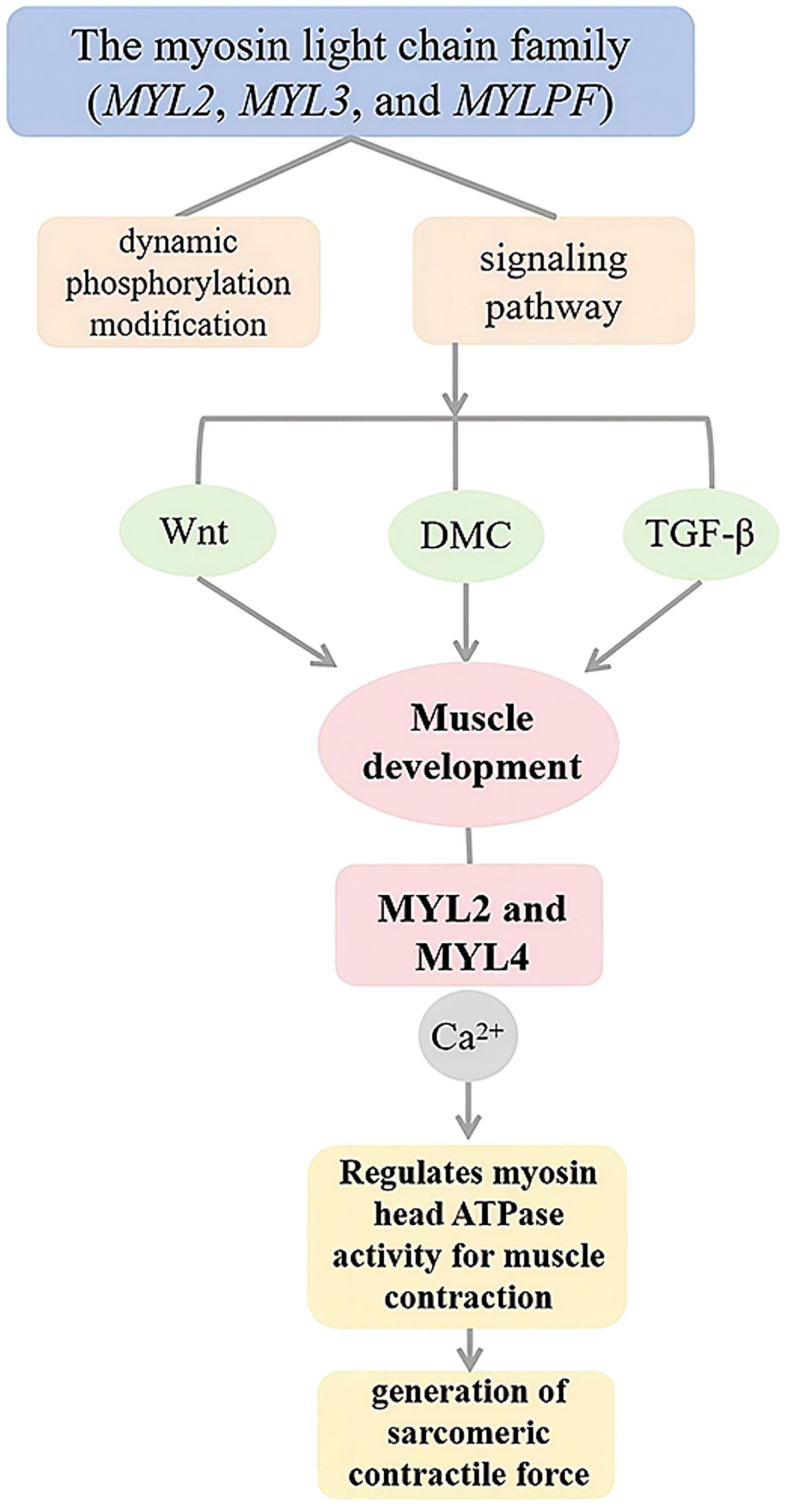
Myosin light chain family genes regulate muscle development through multiple signaling pathways.

Based on the topology analysis of the blue module co-expression network, this study ranked genes by node connectivity and selected the top 10% high-connectivity genes to construct a core regulatory network. By integrating gene expression profile data, four key genes (*MYLPF*, *MYL2*, *PFKM*, and *TCAP*) were found to be positively correlated with network connection strength. Therefore, it was determined that *MYLPF*, *MYL2*, *PFKM* and *TCAP* are the four genes that affect the development of muscle fibers in cashmere goats. Members of the myosin light chain family (*MYL2*, *MYL3*, and *MYLPF*) interact with signaling pathways through dynamic phosphorylation modifications, forming a multi-level regulatory network driving muscle development. These genes are involved in the Wnt, DMC, and TGF-*β* signaling pathways, which significantly impact muscle cell formation and tissue development. *MYL2* and *MYL4* regulate myosin head ATPase activity through their Ca^2+^-binding domains, directly controlling sarcomere contractile force production. Their high expression in embryonic skeletal muscle implies they might affect meat-producing traits by stabilizing structural homeostasis. Studies on chicken leg muscles at different developmental stages showed that proteins related to myofibrogenesis, such as *MYLPF*, *PKM*, *CDKN1B*, *TNNI2*, and *My12*, were upregulated during specific periods (Figure 8). These proteins interact with myocardial contractile and adhesion plaque pathways. Between days 12 and 17 of chicken embryo development, muscle growth-related proteins regulate myoblast proliferation, differentiation, and muscle fiber formation by mediating phosphorylation within key signaling pathways (39). *MYLPF* regulates thin filament activity via Ca^2+^-dependent signaling and modulates muscle function as a skeletal muscle-specific marker of fast-twitch fibers (40). It regulates muscle fiber growth and differentiation in leg muscles during embryonic development and other critical periods (41, 42). *PFKM*, a key glycolytic enzyme, coordinates energy metabolism and muscle fiber hypertrophy by maintaining the ATP/ADP ratio, influencing glycolytic rates and the meat quality of domestic animals (43). Research indicates that the *PFKM* gene is crucial for fat deposition in various pig tissues (44). Tan et al. (43) proposed that *PFKM*, *GAPDH*, and *PKM* might influence muscle fiber type transformation through interactions with differentially expressed proteins, and speculated that *FBP2* could facilitate the shift from slow-to fast-twitch fibers via these interactions (45). Recent studies have revealed that silencing *PFKM* in goat muscle satellite cells alters the expression of 15 meat quality–related genes. These genes are primarily involved in energy metabolism, glucose and lipid metabolism, and cellular structure formation, suggesting that *PFKM* plays a crucial biological role in regulating meat quality (46). *TCAP* is a myofilament protein involved in myofibril assembly, specifically expressed in striated muscle. It serves as a substrate for myosin kinase and binds to the Z1-Z2 region of myosin, providing a binding site (47). Researchers found that *TCAP*, the anchor protein of myoglobin, transfers mechanical stress in the sarcomere via its *β*-folded domain during Z-disk assembly. Its expression level positively correlates with muscle fiber diameter and affects heart and skeletal muscle growth through interactions with other genes (48). In cultured skeletal muscle cells, down-regulation of the *TCAP* gene inhibited myoblast differentiation via RNA interference (49), confirming its close association with skeletal muscle development. Studies have demonstrated that *TCAP* regulates the expression of myostatin (*MSTN*), a known negative regulator of myoblast proliferation and differentiation. Overexpression of *TCAP* leads to a significant reduction in *MSTN* expression (50). These findings further demonstrate that the *TCAP* gene plays a vital role in regulating skeletal muscle growth, differentiation, and regeneration.

As a core member of the myosin light chain family, the *MYL2* gene encodes a protein and has dual functions in mammalian striated muscle. It maintains myofibril stability as a structural protein and regulates muscle contraction through phosphorylation (51). Recent studies have revealed its complex regulatory mechanisms. At the cytodynamic level, via single-cell transcriptome sequencing, Zhan et al. (52) confirmed that *MYL2* shows pulsed expression during skeletal muscle satellite cell differentiation. Its peak expression closely coincides with critical periods of myotube fusion. A comparative study of Tibetan pigs and Yoke pigs showed that the mRNA expression of the *MYL2* gene increased steadily from 30 to 180 days of age, suggesting its role as a key candidate gene in muscle cell proliferation and differentiation (53). In goats, core candidate genes influencing cell adhesion and signaling in myoblasts and myotubes included *CCN2*, *TGFB1*, *MYL2*, and *MYL4* (54). In Black Kite black cattle, high *MYL2* gene expression was observed from the embryonic stage to 2 months old. Real-time fluorescent quantitative PCR indicated that *MYL2* expression peaked at 2 months in the longissimus dorsi muscle, significantly higher than those at 6, 10, or 12 months (*p* < 0.01) (55). Two mutations in the coding sequence (CDS) of the *MYL2* gene were identified in adult Luchuan pigs. These mutations may alter protein phosphorylation patterns, which could affect myosin ATPase activity and subsequently regulate muscle fiber type transformation (11). Research on small-tailed Han sheep also showed that *MYL2* gene phosphorylation activates myosin heavy chain ATPase activity, promoting muscle fiber type conversion and influencing skeletal muscle growth rate and quality (56). After prolonged high-load exercise training, both the mRNA and protein levels of the *MYL2* gene significantly increased in the gluteal medium muscle of Mongolian horses (*p < 0.01*), indicating its role in enhancing endurance-muscle development by improving contraction ability (57).

In summary, the *MYLPF*, *MYL2*, *PFKM*, and *TCAP* genes are thought to influence muscle fiber proliferation at different developmental stages of Inner Mongolia Albas White Cashmere Goats. Their expression levels rise during the late stage of muscle fiber development, suggesting their crucial role in this phase. By analyzing the expression location of the *MYL2* gene in various embryonic stages, we found that it is expressed throughout muscle fiber development. The expression intensity is higher in the late stage of muscle fiber development, with the most obvious positive reaction. Thus, we speculate that the *MYL2* gene may impact the maturation and differentiation of muscle fibers in the late embryonic development stage.

## 5 Conclusion

In summary, we identified a blue co-expression module associated with muscle fiber development, significantly enriched in pathways related to muscle growth. Four key genes-*MYLPF*, *MYL2*, *PFKM*, and *TCAP*-were found to influence muscle fiber development during the late embryonic stage. Notably, *MYL2* exhibited dynamic expression patterns closely aligned with muscle fiber maturation, indicating a potential regulatory role in differentiation. These findings highlight critical genes involved in embryonic muscle development and offer valuable molecular targets for enhancing muscle growth performance and accelerating genetic improvement in goat breeding programs.

## Data Availability

This study involves the RNA-seq original database, accessible at https://www.ncbi.nlm.nih.gov/sra/PRJNA1297081 (BioProject accession number: PRJNA1297081). These data are publicly available and can be freely accessed and used by any researcher.

## Footnotes

^1^ https://www.omicstudio.cn/tool/22

^2^ https://www.omicstudio.cn/tool

^3^ https://string-db.org
